# Self-reported cold sensitivity in patients with traumatic hand injuries or hand-arm vibration syndrome - an eight year follow up

**DOI:** 10.1186/1471-2474-15-83

**Published:** 2014-03-14

**Authors:** Ingela K Carlsson, Lars B Dahlin

**Affiliations:** 1 Department of Hand Surgery, Skane University Hospital, Lund University, Malmö, SE-205 02, Sweden; 2 Department of Clinical Sciences Malmö – Hand Surgery, Lund University, Lund, Sweden

**Keywords:** Cold intolerance, Cold sensitivity, Cold Intolerance Symptom Severity questionnaire, HAVS, SOC, Traumatic hand injury

## Abstract

**Background:**

Cold sensitivity is a common complaint following hand injuries. Our aim was to investigate long-term self-reported cold sensitivity, and its predictors and the importance of sense of coherence (SOC), 8 years after a hand injury as well as in patients treated for Hand Arm Vibration Syndrome (HAVS) during the same time period.

**Methods:**

Responses to the Cold Intolerance Symptom Severity (CISS) questionnaire and the Sense of Coherence (SOC) questionnaire were investigated in hand injured patients (n = 64) and in patients with HAVS (n = 26). The Mann–Whitney U-Test was used to identify significant differences between subgroups. When analysing predictors for cold sensitivity severity, the Spearman rank correlation (*r*_*S*_ coefficient) were used for quantitative predictive variables, Mann–Whitney *U*-Test for dichotomous variables and Kruskal-Wallis Test for multiple categorical data. The Wilcoxon´s signed rank test was used to investigate longitudinal changes in outcome.

**Results:**

There was a significant change in total CISS score for patients with traumatic hand injury, indicating fewer problems with cold sensitivity over time. Symptoms, such as stiffness, weakness and skin colour change on cold exposure, caused fewer problems, but perceived pain/aching and numbness remained unchanged as well as time needed for relief of symptoms on return to a warm environment. The negative impact of cold sensitivity on daily activities and at work was reduced, but problems when engaged in hobbies or when being exposed to cold wintry weather remained unchanged. None of the investigated predictors related to the hand injury were significantly associated with a change in cold sensitivity at the 8-year follow up. In contrast, no significant change in cold sensitivity was noted in the patients with HAVS for any of the situations included in the CISS questionnaire. A lower sense of coherence score correlated significantly with worse cold sensitivity (CISS score) in both patient groups.

**Conclusions:**

The negative impact of cold sensitivity on daily life was reduced for patients with traumatic hand injury, but did not change over time in patients with HAVS. A low SOC is associated with worse cold sensitivity in such groups of patients. Information about relieving strategies should be provided for patients with cold sensitivity.

## Background

Cold sensitivity, described as “an exaggerated or abnormal reaction to cold exposure of the injured part, causing discomfort or the avoidance of cold”, is a common complaint following a variety of hand injuries and diseases [1-10]. The onset of cold-induced symptoms or discomfort is generally reported to occur within the first few months following the injury [11,12]. Contradictory reports exist on whether or not a reduction in discomfort is seen over time and which factor that is linked most strongly to severity and relief of discomfort [8,12-15]. Furthermore, such information has not been presented from different patient populations, such as traumatic injuries and neuropathies. One factor that has to be considered in a long-term follow up is that the reduction of cold-induced symptoms, and the impact cold sensitivity may have on daily life, may result from changes in behaviour, e.g. occupational performance and pattern or access to other coping strategies [16,17]. How patients view their life and see the world as comprehensible, manageable and meaningful (sense of coherence – SOC) may also facilitate the adaptation process [18-20].

The pathophysiology behind the complex phenomenon of cold sensitivity remains unclear, thus, a multifactor aetiology, including bony, vascular and neural components, is suggested, which was previously confirmed by a high correlation between a Hand Injury Severity Score (HISS), indicating more severely injured hands, and worse self-reported cold sensitivity (CISS score) [21].

When defining symptoms and signs and clarifying the impact of cold sensitivity on daily life, it is important to use valid and reliable questionnaires, such as the Cold Intolerance Symptom Severity (CISS) questionnaire [14,22]. A cut-off value for abnormal self-reported cold sensitivity in a normal Swedish population has been established previously. It suggests that a total CISS score above 50 indicates abnormality [21]. Our aim was to investigate long-term, i.e. self-reported cold sensitivity 8 years after the occurrence of the hand injury as well as in patients treated for Hand Arm Vibration Syndrome (HAVS) during the same time period. Furthermore, we also looked for any predictors associated with some change in cold sensitivity over time for patients with traumatic hand injury, as well as the association between patients’ sense of coherence and cold sensitivity.

## Method

### Study groups and methods

During the cold season of 2011 the Swedish version of the Cold Intolerance Symptom Severity (CISS) questionnaire and the 13-item condensed Sense of Coherence (SOC) questionnaire [18,22] was sent to all patients (n = 118) with a registered diagnosis (patient register at University Hospital Malmö) of a digital or midcarpal amputation (n = 52), a traumatic nerve lesion (n = 36) or HAVS (n = 30), excluding those below 18 years of age. The patients with traumatic hand injury were treated during January-November 2003 and the patients with HAVS during 2002–2003 and responded to the Cold Intolerance Symptom Severity (CISS) questionnaire in 2004. The patients with a traumatic injury were surgically treated at the department following the decision of the individual treating surgeon. The severity of the hand injury for each patient was defined by the hand injury severity score (HISS) [23]. Since there were no significant differences in cold sensitivity (CISS 4–100) between patients with amputation injuries and those with nerve injuries either at 1 year or at 8 years follow up, these patients were subsumed in a single group (traumatic hand injuries). The diagnosis of HAVS was based on a history of vibration-induced symptoms, for example white fingers, and/or sensorineural symptoms, with or without impaired vibrotactile sense and neurophysiological findings, supporting the presence of HAVS, but excluding other causes of neuropathy. All patients and subjects were from the southern part of Sweden which has a mean temperature of 0°C during the cold season ( http://www.smhi.se). One written reminder was sent out to the patients. Nine patients with a traumatic hand injury and one patient with HAVS had passed away since the initial survey in 2004 leaving a total of 108 patients. Ninety patients (83%) (amputation n = 37, nerve-injury n = 27, HAVS n = 26) responded. No discernable differences were found between the responders and non-responders with respect to age and gender. There was an internal dropout for the CISS questionnaire (traumatic hand injury, n = 4, HAVS, n = 2) in that not all patients answered all questions included in the total CISS score at both one and 8 years follow up, leaving at total of 60 patients with traumatic hand injury and 24 patients with HAVS.

All participants gave their informed consent to participating in the study, which was approved by the Ethics Committee, Faculty of Medicine, Lund University.

### Data analyses

Results are presented as median (range). The Mann–Whitney U-Test was used to identify significant differences between subgroups. When analysing predictors for cold sensitivity severity (CISS score) the Spearman rank correlation (*r*_*S*_) coefficient were used for quantitative predictive variables, the Mann–Whitney *U*-Test for dichotomous variables and the Kruskal-Wallis Test for multiple categorical data. The Wilcoxon´s signed rank test to investigate the longitudinal changes in outcome. *P*-values of less than 0.05 were considered significant. Data were analysed using the SPSS software package, version 12.0.1.

## Results

The characteristics of the subjects and patients are described in Table 1. The patients with traumatic hand injury had a median HISS score of 77 (10–305) [21]. Fifteen of the 26 patients with HAVS had according to the Stockholm Workshop scale vibration-induced white fingers, 23 of 26 patients had sensorineural symptoms and 20 of 26 had both white fingers and sensorineural symptoms (Table 1). Twenty-two of the 26 patients had an impaired vibrotactile sense. At the present follow-up, fourteen patients with HAVS were retired either due to old age (n = 7) or received early retirement pension because of disability or sickness (n = 7). Twelve patients worked at the time for the investigation, but nine of these patients had changed work or work tasks during the 8-year follow up to eliminate vibration exposure. The remaining three patients with HAVS had an average of 2 hours/day of vibration exposure with tools, such as grinding machines and power drills. All patients avoided vibration exposure during leisure with the exception of minor exposure when e.g. cutting the lawn or using an electric drill.

**Table 1:** Characteristics of patients with traumatic hand injury and HAVS

**Parameter**	**Traumatic hand injury n =** **64**	**HAVS n =** **26**
Gender (male/female)	50/14	24/2
Age at 8 year follow up^1,2^	51 (28–86)	61 (31–73)
Smoker (yes/no)	14/50	4/22
Time since injury (years) at 8 year follow up^3^	8.25 (7.75–9)	-
Years of vibration exposure at 1 year follow up^1^	-	30 (4–46)
CISS score (abnormal/normal)^4^	17/43	16/8
DASH score at 1 year follow up (0–100)^5,1,6^	23 (2–60)^5^	34 (5–70)^5^
Sense of coherence (SOC, range 13–91) score at 8 year follow up^3,7^	74 (28–91)	67 (50–91)
HISS at injury^1,8^	77 (10–305)	-
Vibration-induced white fingers (VWF)^9^	-	15/26
Stage		
0		11^9^
1		5
2		7
3		3
4		0
Sensorineural symptoms	-	23/26
Stage		
0		3
1		8
2		7
3		8
Both VWF and sensorineural symptoms^10^	-	20/26
Impaired vibrotactile sense^11^	-	22/26

^1^Median (IQR).

^2^Patients with HAVS were significantly older than patients with traumatic hand injuries (p = 0.004).

^3^Median (range).

^4^Internal drop out: traumatic hand injury (n = 4), HAVS (n = 2). CISS score >50 = abnormal self-reported cold sensitivity [21].

^5^Disability of the Arm, Shoulder and Hand (DASH). 0 = no disability, 100 = most severe disability [24].

^6^Patients with HAVS had significantly higher scores indicating more severe disability (p = 0.001).

^7^The 13-item scale. The scores on each item ranges from 1 (never) to 7 (very often). A high score indicates a strong SOC [18].

^8^Hand Injury Severity Score [23].

^9^Stockholm Workshop scale [25]. 8 out of 11 patients had cold sensitivity without blanching of skin (i.e. equal to 0.5 in a modified Stockholm Workshop scale, VWF). Staging is based on the highest stage on the most injured hand.

^10^Patients having both a VWF score ≥ 0.5 and sensorineural score > 0.

^11^Sensibility index (SI) < 0.8 in at least one finger [26]. SI-index is a measure of vibrotactile sense and is a ratio of the integrated area below a test curve and a corresponding age reference population. An index > 0.8 is regarded as an indication of abnormality [27].

### Change in self*-*reported cold sensitivity (CISS score) between 1 and 8 year follow up

**Figure 1:**
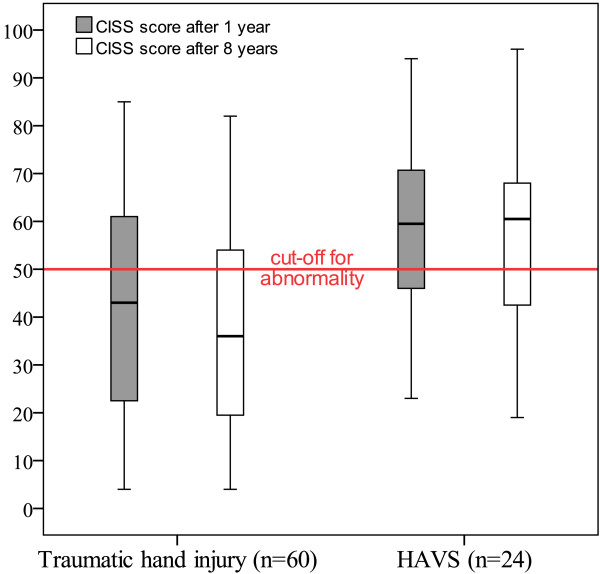
**CISS score 1 year postoperatively and 8 years postoperatively in patients with traumatic hand injury (p =** **0.001, n =** **60) or HAVS (p =** **0.5, n =** **24).** 17/60 patients with traumatic hand injury and 16/24 patients with HAVS had abnormal CISS score after 8 years. The corresponding numbers at 1 year follow up was 39/86 and 21/28 respectively.

**Figure 2:**
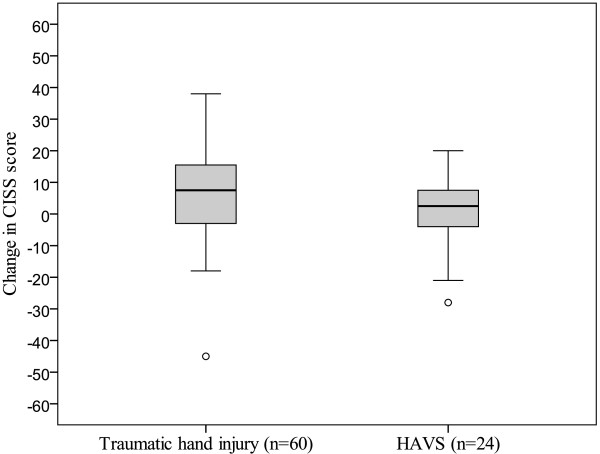
**Change in CISS score between 1 year postoperatively and 8 years postoperatively in patients with traumatic hand injury (n =** **60, p =** **0.001) or HAVS (n** = **24, p =** **0.50).**

There was a significant change in the total CISS score for patients with traumatic hand injury (p = 0.001, n = 60), indicating less problems with cold sensitivity over time, in contrast to those with HAVS (p = 0.50, n = 24), (Figures 1 and 2, Table 2). Patients with traumatic hand injuries experienced significantly less problems over time with stiffness, weakness and skin colour change on cold exposure, but not with pain, aching, numbness and swelling (Table 3). The only symptom that patients with HAVS experienced significantly less problems with was weakness on cold exposure (Table 3).

**Table 2:** **Responses to the CISS questionnaire at 1 year and 8 years follow**-**up in patients with traumatic hand injury or HAVS**

**CISS questionnaire**^**1**^	***Score***	**Traumatic hand injury**				**HAVS**			
		**n**	**1 year**^**2**^	**8 year**^**2**^	**p-value**^**3**^	**n**	**1 year**^**2**^	**8 year**^**2**^	**p-value**^**3**^
Total CISS score	4-100	60	45 (4–85)	36 (4–82)	**0.001**	22	60 (23–94)	60 (19–96)	0.50
2. How often do you experience these symptoms?		60	8 (2–10)	6 (2–10)	0.06	22	8 (2–10)	8 (2–10)	1.0
- Continuously/all the time	10								
- Several times a day	8								
- Once a day	6								
- Once a week	4								
- Once a month or less	2								
3. When you develop cold-induced symptoms, on your return to a warm environment are the symptoms relieved		60	6 (2–10)	6 (2–10)	0.31	22	6 (2–10)	6 (2–10)	1.0
- Within a few minutes	2								
- Within 30 minutes	6								
- After more than 30 minutes	10								
4. What do you do to ease or prevent your symptoms occurring?		62	4 (0–10)	4 (0–10)	0.71	22	5 (0–10)	4 (2–10)	0.67
- Take no special action	0								
- Keep hand in pocket	2								
- Wear gloves in cold weather	4								
- Wear gloves all the time	6								
- Avoid cold weather/stay indoors	8								
- Other	10								
5. How much does cold bother your injured hand in the following situations?									
- Holding a glass of ice water	0–10	62	3 (0–10)	2 (0–10)	**0.008**	22	5 (0–10)	5 (0–10)	0.74
- Holding a frozen package from the freezer	0–10	60	5 (0–10)	3 (0–10)	**0.004**	22	7 (0–10)	7 (1–10)	0.48
- Washing in cold water	0–10	62	4 (0–10)	2 (0–10)	**0.02**	22	7 (0–10)	6 (1–10)	0.24
- When you get out of a hot bath/shower with the air at room temperature	0–10	62	0 (0–8)	0 (0–8)	**0.005**	22	3 (0–10)	2 (0–10)	0.31
- During cold wintry weather	0–10	62	7 (0–10)	5 (0–10)	0.06	22	8 (4–10)	8 (2–10)	0.18
6. Please state how each of the following activities have been affected as a consequence of cold-induced symptoms in your injured hand and score each.									
- Domestic chores	0–4	62	1 (0–4)	0 (0–4)	**0.044**	22	2 (0–3)	2 (0–4)	0.72
- Hobbies and interests	0–4	62	2 (0–4)	1 (0–4)	0.16	24	3 (0–4)	2 (0–4)	0.63
- Dressing and undressing	0–4	62	0 (0–4)	1 (0–4)	0.17	22	1 (0–4)	1 (0–4)	0.79
- Tying your shoe laces	0–4	62	1 (0–4)	1 (0–4)	0.06	22	2 (0–4)	1 (0–4)	0.22
- Your job	0–4	62	2 (0–4)	2 (0–4)	**0.04**	22	3 (0–4)	2 (0–4)	0.37

^1^Question no 1 is not included in the total CISS score. See Table 3 for details.

^2^Median values (range).

^3^Wilcoxon’s signed rank test. Bold = statistical significance.

**Table 3:** **Perceived symptoms on cold exposure at 1 and 8 years follow**-**up in patients with traumatic hand injury or HAVS**

**Perceived symptoms on cold exposure**^**1**^	***Score***	**Traumatic hand injury**				**HAVS**			
		**n**	**1 year**^**2**^	**8 years**^**2**^	**p-value**^**3**^	**n**	**1 year**^**2**^	**8 years**^**2**^	**p-value**^**3**^
Pain	*0*-*10*	54	3 (0–6)	3 (0–5)	0.46	18	6 (3–8)	6 (2–7)	0.25
Numbness	*0*-*10*	51	4 (1–6)	3 (0–6)	0.07	18	6 (4–8)	7 (5–8)	0.87
Stiffness	*0*-*10*	53	5 (2–7)	4 (1–6)	**0.001**	22	7 (4–8)	6 (4–8)	0.53
Weakness	*0*-*10*	52	5 (1–7)	3 (1–6)	**0.004**	19	7 (4–8)	5 (4–7)	**0.04**
Aching	*0*-*10*	57	3 (0–7)	2 (0–5)	0.12	19	6 (4–8)	7 (4–8)	0.85
Swelling	*0*-*10*	58	0 (0–2)	0 (0–1)	0.18	21	0 (0–4)	2 (0–5)	0.11
Skin colour change	*0*-*10*	53	3 (0–7)	2 (0–5)	**0.001**	17	6 (4–8)	5 (3–8)	0.14

^1^Question no 1 in the CISS questionnaire: “Which of the following symptoms of cold intolerance do you experience in your injured limb on exposure to cold?” (0 = no symptoms/trouble at all and 10 = the most severe symptoms/trouble you can possibly imagine).

^2^Median values (q_1_–q_3_).

^3^Wilcoxon’s signed rank test. Bold = statistical significance.

Patients with traumatic hand injuries also experienced less problems when exposed to cold during daily activities, e.g. when holding a glass of ice water or frozen package, when being exposed to cold water or temperature shifts, such as getting out of a hot bath/shower with the air at room temperature. The negative impact of cold-induced symptoms during domestic chores and at work was also improved. There was no significant change in the time needed to experience relief of symptoms on return to a warm environment, nor was any improvement noted when engaged in hobbies or interests or when being exposed to cold wintry weather. For patients with HAVS no significant change in cold sensitivity was noted for any of the situations included in the CISS questionnaire (Table 2).

### Predictors for change over time in self*-*reported cold sensitivity for traumatic hand injuries

None of the factors listed in Tables 4 and 5 were significantly associated with change in cold sensitivity at 8-year follow up. The presence of bone injury, a larger number of repaired vessels, the use of vascular grafts and a high Hand Injury Severity Score (HISS) were linked to worse CISS scores at one year follow up [21], but failed to show any significant association for change in self reported cold sensitivity in the present long term follow up.

**Table 4:** **Predictors** (**quantitative variables**) **of change in self**-**reported cold sensitivity** (**CISS score 4**–**100**) **among patients with traumatic hand injuries**

**Continuous variables**	**Change in CISS score (4–100) (n =** **59–60)**	
	**r**_**s**_^**1**^	**p-value**
Age	0.17	0.18
Time after injury (months)	−0.10	0.44
HISS score^2^	0.12	0.38
Number of repaired nerves	−0.02	0.90
Number of injured vessels	0.04	0.75
Number of repaired vessels	0.02	0.88
Number of surgical sessions	0.04	0.79

^1^Spearman rank correlation test.

^2^Hand Injury Severity Score, higher score indicates worse anatomical damage.

**Table 5:** **Predictors** (**categorical variables**) **of change in self**-**reported cold sensitivity** (**CISS**; **score range 4**–**100**) **among patients with traumatic hand injuries**

**Multiple and dichotomous predictors**		**Change in total CISS score (4–100)**		
		**n**	**Md (q1, q3)**	p-value*
Gender	Male	46	5 (−3, 16)	0.75
	Female	14	10 (−5, 14)	
Smoking	No	46	10 (−3, 17)	0.23
	Yes	14	2 (−4, 12)	
Injured side	Dominant	27	3 (−3, 13)	0.37
	Non dominant	31	9 (−3, 18)	
	Bilateral	2	−15 (−45)	
Injured digit(s)	Single digit	17	6 (−5, 14)	0.918
	Multiple digits	17	9 (−3, 17)	
Level of injury	Distal to the MCP joint level	30	9 (−3, 16)	0.77
	Mid palm and dorsal hand	19	1 (−6, 17)	
	Proximal hand	4	5 (−3, 22)	
	Wrist and forearm	7	9 (1, 14)	
Type of injury	Sharp	32	3 (−3, 16)	0.63
	Laceration	20	10 (3, 15)	
	Crush	8	7 (−11, 29)	
Soft tissue damage	No	33	3 (−6, 14)	0.06
	Yes	27	10 (2, 17)	
Soft tissue repair	None	8	8 (−15, 17)	0.37
	Skin suture	38	2 (−3, 15)	
	Split skin	12	12 (7, 16)	
	Flap	2	13 (2)	
Bone injury	No	30	2 (−5, 16)	0.19
	Yes	30	10 (−2, 16)	
Osteosynthetic material	None	37	2 (−3, 17)	0.53
	Pins/Wire	13	10 (5, 16)	
	Screws or plate	10	12 (−6, 16)	
Removal of osteo-synthetic material	No	2	25 (11)	0.19
	Yes	14	12 (9, 14)	
Tendon injury	No	20	3 (−2, 17)	0.97
	Yes	40	9 (−3, 15)	
Vascular grafts	No	49	3 (−3, 15)	0.16
	Yes	10	13 (7, 17)	
Nerve injury	No nerve injury	5	3 (−5, 18)	0.39
	Median nerve	10	−5 (−6, 13)	
	Ulnar nerve	5	17 (9, 20)	
	Median and ulnar or multiple nerves	3	−1 (−45)	
	Radial nerve	5	9 (−11, 12)	
	One digital nerve	5	14 (−2, 20)	
	Two or multiple digital nerves or common digital nerve	26	10 (−3, 15)	
Nerve injury	Complete	46	8 (−3, 15)	0.26
	Partial	5	−3 (−6, 11)	
	Contusion	6	20 (14, 31)	
Repaired nerve	No	36	8 (−3, 15)	0.83
	Yes	14	3 (−3, 14)	
Revascularisation	No	51	4 (−3, 15)	0.60
	Yes	9	12 (−6, 19)	
Replantation	No	48	4 (−3, 17)	0.88
	Yes	12	10 (−3, 13)	
Post operative pain relief	None	4	6 (−13, 10)	0.87
	Oral	25	9 (−3, 17)	
	Injection	23	6 (2)	

*Mann–Whitney U-Test was used for dichotomous variables and Kruskal-Wallis was used for variables with multiple categories.

### Self reported cold sensitivity and sense of coherence

A lower sense of coherence score correlated significantly with worse cold sensitivity (CISS score) in both patient groups at the 8-year follow up (trauma group: r^s^ = 0.31, p-value 0.016, HAVS group: r^s^ = 0.47, p-value 0.027). However, there was no significant relationship (p-value = 0.26) between the sense of coherence score and change in cold sensitivity between the 1 and 8 year follow up. In addition, there was no significant difference in the sense of coherence score between patients with traumatic hand injury or HAVS (p = 0.21).

## Discussion

Our results showed a significant long-term improvement (total CISS score) in self-reported cold sensitivity in patients with traumatic hand-injury. However, there was no improvement in pain levels on cold exposure or time needed to feel relief of symptoms on return to a warm environment, nor was any improvement noted when engaged in leisure activities or when being exposed to cold wintry weather. None of the predictors were significantly linked to any change in cold sensitivity in patients with traumatic hand-injury. For patients with HAVS no significant change was noted in total CISS score or in any of the included questions in the CISS questionnaire. A lower sense of coherence score correlated significantly with worse cold sensitivity (total CISS score) in both patient groups at 8-year follow up.

There are few long-term studies on the prolonged effect of cold sensitivity and among them the results are contradictory. Furthermore, the use of different outcome measures, such as single yes or no questions, verbal or numeric rating scales or more extensive questionnaires, make comparisons difficult. Many studies on nerve injuries indicate that there is no significant change in cold sensitivity over time for the majority of patients [8,11]. For amputation injuries, with or without replantation, some studies report weakened problems with time, while others report very little improvement [28-30]. Nancarrow et al. reported unchanged problems at 5-year follow up for a mixed population of traumatic hand injuries, which is in line with the results of Gustafsson et al. at a 10-year follow up. Riaz et at concluded that around half of a patient group with flexor tendon injuries had persistent problems after 10.6 years [7,13,31]. In a prospective cohort study of all patients with acute trauma to the hand or forearm, Craigen et al. found that the degree of cold sensitivity increased rapidly up to three months, then plateaued and remained unchanged for at least eight months. A reduction in discomfort over a period of three years was most pronounced in patients with severe symptoms [12]. This finding is in contrast with Povlsen et al., who did not find any improvement in severely affected patients 12 years after digital replantation, but some improvement in patients with initial moderate discomfort [15]. Our findings support an improvement in cold sensitivity for patients with traumatic hand injuries as measured by the total CISS score. Thus, important information about e.g. pain levels (CISS question no 1) on cold exposure, which is not included in the total score, did not change significantly over time, nor did the time needed for relief of symptoms on return to a warm environment or discomfort when engaged in leisure activities or during cold wintry weather (CISS question no 3, 5 and 6) Although the CISS questionnaire, with its total score, is considered reliable and the content and construct validity has been good [22], our findings highlight the importance to view each individual question since the included questions in CISS is not weighted and important information otherwise will be lost.

In our previous study, we found that a high HISS score indicating more severely injured hands correlated with abnormal cold sensitivity (CISS score > 50), but not with any change in CISS score at the present 8-year follow up [21]. Thus, the severity of the hand injury did not predict change in cold sensitivity over time. We have no explanation for the lack of predictors, but one may speculate that there is maturation over time in such injuries and possibly also a component of coping. A limitation of the study may also be that there are a limited number of patients. There may be a risk that we do not see any effect of certain variables.

Surprisingly, there were a few patients with HAVS that were still exposed to vibrations. The general recommendation for this patient group with problems of e.g. cold sensitivity is that vibration exposure should be avoided. Furthermore, our long-term follow up of patients suffering from HAVS clearly showed that they experienced unchanged problems on cold exposure, which is interesting in view of the pathophysiology with a neural component as the cause of the symptoms. In contrast, patients with diabetes and a carpal tunnel syndrome experience remaining symptoms with cold sensitivity after carpal tunnel release at one year, but not at five years after the release [32,33]. Thus, different types of neuropathies may have diversity in their development and or their adaptation to cold sensitivity, or both.

Thus, the improvement after 8 years seen in our patients with a traumatic hand injury may be a result of an actual improvement or a result of an adaptation process. Such an adaptation process has been previously described and the importance of access to relieving strategies for hand injured patients has been highlighted [17]. These strategies could e.g. be use of heating remedies, proper gloves, overall warm general clothing and a choice of tools with a surface layer causing less cold exposure. To put injured hand in armpit, to exercise or actively move the affected hand, to change grip pattern or hide the injured hand when opening the refrigerator, to increase indoor temperature, turning off ventilation/air conditioning or avoiding cold water may be other alternatives. At 1 year follow up abnormal cold sensitivity (CISS score > 50) was seen in 45% and 75% of patients with traumatic hand injury and HAVS respectively [1]. The corresponding percentage of abnormality was for those responded at the present 8-year follow up, 28% and 62% respectively. The majority of the included questions in the total CISS score focus on the impact of cold sensitivity on activities in daily life. Our result mirrors an increased ability for patients with traumatic hand injury to master challenges in daily life caused by cold exposure. Adaptation strategies as described above probably played an important role in that process. Especially, since symptoms, such as pain, aching and numbness, did not improve significantly; neither did the time needed to experience relief on return to a warm environment in our patient groups.

A novel finding in this study was the significant relationship between worse self-reported cold sensitivity (CISS score) and a lower sense of coherence in both patient groups. For more than 20 years ago the American-Israeli medical sociologist Aaron Antonovsky introduced his salutogenic theory 'sense of coherence' as a global orientation to view the world, claiming that the way people view their life has a positive influence on their health. Sense of coherence explains why people in stressful situations stay well and even are able to improve their health. The person’s ability or disposition to see the world as comprehensible, manageable and meaningful is therefore reflected in the SOC questionnaire [18,34]. How patients with a severe hand injury adapt to the consequences of cold sensitivity in daily life may very well be linked to an ability to view a new situation in life according to these perspectives. This seems to be especially important when severe cold sensitivity result in changed life roles e.g. a work role or the role of being a parent. The alterations in or loss of work tasks or the ability to take part in leisure activities with spouse and children has previously been described to cause frustration and distress and even influence self-image [17].

## Conclusion

We conclude that the negative impact of cold sensitivity on activities in daily life improved during the 8-year follow up for patients with traumatic hand injury despite unchanged problems with symptoms, such as pain/aching and numbness on cold exposure, and that the time needed to experience relief on return to a warm environment did not improve. In contrast, patients with HAVS did not show any significant improvement. Interestingly, a low SOC was associated with worse cold sensitivity (CISS score) in such groups of patients. Information should be provided about relieving strategies to assist patient’s adaptation process.

## Competing interests

The authors declare that they have no competing interests.

## Authors’ contributions

IC designed the study and performed the statistical analysis. IC and LD interpreted the findings and drafted the manuscript. Both authors read and approved the final manuscript.

## Pre-publication history

The pre-publication history for this paper can be accessed here:

http://www.biomedcentral.com/1471-2474/15/83/prepub
